# MOF-Derived Co_3_O_4_ Polyhedrons as Efficient Polysulfides Barrier on Polyimide Separators for High Temperature Lithium–Sulfur Batteries

**DOI:** 10.3390/nano9111574

**Published:** 2019-11-06

**Authors:** Zhenfang Zhou, Yue Li, Tingting Fang, Yufeng Zhao, Qingjie Wang, Jiujun Zhang, Zhongfu Zhou

**Affiliations:** 1 School of Materials Science and Engineering, Shanghai University, Shanghai 200444, China; zzf890225@163.com (Z.Z.); liyuejanewatson@163.com (Y.L.); ftt550627949@sina.com (T.F.); 2 Institute for Sustainable Energy, College of Science, Shanghai University, Shanghai 200444, China; jiujun.zhang@i.shu.edu.cn; 3 State Key Laboratory of Advanced Chemical Power Sources, Guizhou Meiling Power Sources Co. Ltd., Zunyi 563003, Guizhou, China; wqj3401@163.com; 4 Department of Physics, Aberystwyth University, Aberystwyth SY23 3BZ, UK

**Keywords:** energy storage systems, lithium sulfur batteries, functional separators, high temperature operation, polyimide

## Abstract

The incorporation of highly polarized inorganic compounds in functional separators is expected to alleviate the high temperature safety- and performance-related issues for promising lithium–sulfur batteries. In this work, a unique Co_3_O_4_ polyhedral coating on thermal-stable polyimide (PI) separators was developed by a simple one-step low-temperature calcination method utilizing metal-organic framework (MOF) of Co-based zeolitic-imidazolate frameworks (ZIF-Co) precursors. The unique Co_3_O_4_ polyhedral structures possess several structural merits including small primary particle size, large pore size, rich grain boundary, and high ionic conductivity, which endow the ability to adequately adsorb dissolved polysulfides. The flexible-rigid lithium-lanthanum-zirconium oxide-poly(ethylene oxide) (LLZO-PEO) coating has been designed on another side of the polyimide non-woven membranes to inhibit the growth of lithium dendrites. As a result, the as-fabricated Co_3_O_4_/polyimide/LLZO-PEO (Co_3_O_4_/PI/LLZO) composite separators displayed fair dimensional stability, good mechanical strength, flame retardant properties, and excellent ionic conductivity. More encouragingly, the separator coating of Co_3_O_4_ polyhedrons endows Li–S cells with unprecedented high temperature properties (tested at 80 °C), including rate performance 620 mAh g^−1^ at 4.0 C and cycling stability of 800 mAh g^−1^ after 200 cycles—much better than the state-of-the-art results. This work will encourage more research on the separator engineering for high temperature operation.

## 1. Introduction

Characterized by high energy density and good cycling stability, lithium ion batteries have been intensively considered as future electric power supplies for electrical vehicles (EVs) [1,2]. However, the uncertainty of EVs application environments, such as cold winter and hot summer, accompanied with long-distance driving, sets higher requirements for the power batteries, which should possess good weatherability, high temperature tolerance, and high energy density [3,4,5]. It is generally known that both the electrode materials and the separators play vital roles in achieving safe and high energy density batteries. Cathode materials with multi-electron charge transfer processes could deliver higher specific capacity and thus higher energy density [6,7,8]. Lithium sulfur batteries composed of lithium metal anode and sulfur cathode have been recognized as one of such battery systems due to their extremely high theoretical energy density (2600 Wh kg^−1^, almost 7 times higher than that of typical layered oxide cathodes) [9,10]. During the past decade, great progress has been made in the development of room temperature lithium–sulfur batteries [11,12]. However, the safety and high temperature application of lithium–sulfur batteries are still facing serious challenges due to the electronic insulation of elemental sulfur, the dissolution and shuttle effect of polysulfide, and the growth of lithium dendrite.

Searching for functional separators with high thermal stability and polysulfide adsorption-anchoring capability is one of the most effective strategies to address the safety and high temperature performance-related issues for lithium–sulfur batteries [12,13]. Coating functional materials with high electronic conductivity and high charge polarization could endow the separators with the ability to physically/chemically anchor the soluble polysulfide species, leading to higher battery performances for room temperature lithium sulfur batteries [12,14]. Carbon-based materials are widely used in separator coating materials for lithium sulfur batteries due to their multifarious advantages of light weight, high conductivity, diversity, and easy-processability [15,16,17]. For instance, various porous carbon materials with adjustable pore size could capture dissolved polysulfide species via space confinement effects and van der Waals interaction [18,19]. The high electronic conductivity of these carbon-based materials also facilitates the further conversion of captured polysulfide species [15,17]. However, due to the weak interaction of van der Waals force between the non-polar carbon materials and the polysulfide species, the shuttle effect inevitably occurs in lithium sulfur battery systems using the carbon-based material-modified separators [13,14,20]. Recently, inorganic compounds such as TiO_2_, MnO_2_, MoS_2_, and VN have been proved to show stronger polysulfide bonding than normal carbon-based materials as a result of the formation of strong chemical interactions between these compounds and polysulfide species [13,20,21,22]. Therefore, when these polar inorganic compounds are served as host materials or separator coating materials, room temperature lithium–sulfur batteries exhibit better cycling performance [13,20,21,22].

Nevertheless, most of polar inorganic materials are semiconductors in nature, which show rather low electronic conductivity and dramatically hinder the further transformation of the adsorbed polysulfides [23,24]. In addition, the commonly-used transition-metal oxides have smaller pore structure and are easy to agglomerate during the preparation processes. These facts inevitably result in a decreased proportion of surface atoms, the greatly restricted transformation of lithium ions, and the insufficient utilization of the exposed surface structure of polar transition metal oxides. Therefore, there is still an urgent task to search for suitable polar inorganic materials with appropriate chemical composition, good electronic conductivity, desirable pore size, and optimized micro/nano-dimensional structures by large-scalable methods to address the shuttle effect of polysulfides for rechargeable lithium sulfur batteries.

As a kind of promising polar inorganic compound, Co_3_O_4_ nanostructures have been proved to show strong adsorption towards the polysulfide species and thus be suitable as room temperature sulfur hosts [25]. Ji et al. have prepared the bamboo-like Co_3_O_4_ nanostructures by hydrothermal method as sulfur hosts, which exhibited an initial specific capacity of 1100 mAh g^−1^ at 0.1 C [26]. Even after 300 cycles at 1 C, the Co_3_O_4_ nanostructures still delivered a moderate specific capacity of 796 mAh g^−1^. Wang et al. [27] presented the sulfur/Co_3_O_4_ composite nanotubes by melting-infiltration method and studied their lithium storage performance at room temperature. The above demonstrations showed that the Co_3_O_4_ nanostructure possessed strong adsorption–anchoring function for polysulfide species and the fabricated lithium–sulfur batteries exhibited excellent cyclic stability, verifying the reliability and viability of the Co_3_O_4_ nanostructures. In spite of these achievements, the as-prepared nanostructure generally showed smaller pore size structure and larger initial particle size, leading to the incomplete utilization of active surface atoms. In addition, the constructed lithium–sulfur batteries do not display the best electrochemical performance and their high temperature performances are still unexplored to date.

In this work, a unique Co_3_O_4_ polyhedral coating on thermal-stable polyimide separators has been developed by a simple one-step low-temperature calcination method utilizing a metal-organic framework of ZIF-Co precursors. The unique Co_3_O_4_ polyhedral structures are characterized by small primary particle size, large pore size, rich grain boundary, and high ionic conductivity. These structural merits endow the Co_3_O_4_ polyhedral structures with the ability to adequately adsorb dissolved polysulfides, which significantly improve the cyclic stability of lithium–sulfur even under high temperature conditions. To inhibit the growth of lithium dendrites, the flexible-rigid LLZO-PEO coating has been designed on another side of the polyimide non-woven membranes. The as-fabricated Co_3_O_4_/PI/LLZO composite separators displayed dimensional stability, good mechanical strength, flame retardant properties, and excellent ionic conductivity. The experimental data show that the lithium–sulfur batteries assembled with Co_3_O_4_/PI/LLZO composite separators exhibit excellent charge–discharge capacity and cycle stability at elevated temperature.

## 2. Materials and Methods

Separator fabrication: Preparation of the Co_3_O_4_-X/PI/LLZO separator (X: 350 or 500): The ZIF-Co polyhedron was synthesized by facile chemical precipitation method. Firstly, 13.7 mmol Co(NO_3_)_2_·6H_2_O and 63.9 mmol 2-methylimidazole were respectively dissolved into 400 mL methanol with vigorous stirring for 1.0 h. Then, the cobalt nitrate solution was poured into the 2-methylimidazole solution, stirred for 5 min, and the homogeneous solution was rested at room temperature for 24 h. Finally, the ZIF-Co purple precipitated powder was washed three times with methanol and ethanol and dryed in an oven at 60 °C. The ZIF-Co polyhedron was used as a sacrificial template precursor, which was calcined at 350 or 500 °C for 2 h at a ramp rate of 3 °C min^−1^ in air. The obtained samples were recorded as Co_3_O_4_-350 polyhedron and Co_3_O_4_-500 polyhedron, respectively. Then, both the Co_3_O_4_-350 polyhedron and Co_3_O_4_-500 polyhedron were respectively severed as active materials, which were mixed with acetylene black and poly(vinylidene fluoride) (PVDF) at a mass ratio of 5:4:1 in N-methyl pyrrolidone solution to form a homogeneous slurry. Finally, the slurry was coated on one side of the non-woven PI film with a scraper by a typical blade-coating method and dried in a vacuum oven at 60 °C for 10 h to obtain the Co_3_O_4_-350/PI and Co_3_O_4_-500/PI film, respectively. The LLZO-PEO white emulsion synthesized by mixing the LLZO and PEO at a mass ratio of 1:10 in acetonitrile solvent is coated on the other side of bot the Co_3_O_4_-350/PI film and Co_3_O_4_-500/PI film, and dried for 6 h in a vacuum oven at 60 °C to obtain the Co_3_O_4_-350/PI/LLZO separator and the Co_3_O_4_-500/PI/LLZO separator, respectively.

Material characterizations: The crystal phase and micro-structure of the Co_3_O_4_-350 polyhedron and the Co_3_O_4_-500 polyhedron were measured and analyzed by X-ray diffraction with Cu K_a_ radiation (XRD, Rigaku D-max-gA, λ = 1.54178 Å, Tokyo, Japan). The morphology and particle size of the Co_3_O_4_-350 polyhedron and the Co_3_O_4_-500 polyhedron were characterized by the scanning electron microscopy (SEM, JSM 6700F, Tokyo, Japan). The fine microstructures of the Co_3_O_4_-350 polyhedron and the Co_3_O_4_-500 polyhedron were characterized by transmission electron microscopy (TEM, JEOL 2100F, Tokyo, Japan). The lattice structures of the Co_3_O_4_-350 polyhedron and the Co_3_O_4_-500 polyhedron were characterized by high resolution transmission electron microscopy (HRTEM). The N_2_ adsorption–desorption curves and pore size distribution of the Co_3_O_4_-350 polyhedron and the Co_3_O_4_-500 polyhedron were measured by a specific surface analyzer. 

Cell fabrication and electrochemical measurements: The typical sulfur–carbon nanotubes (S@CNTs) composite was utilized as cathode, which was synthesized according to our previous work [28]. For each cell, the sulfur cathode was tailored into a cylindrical electrode and the average sulfur loading was about 2.5 mg cm^−2^. The lithium metal foil was served as the anode. For testing at room temperature, the electrolyte was the 1,2-dimethoxyethane/1,3-dioxolane (DME/DOL; 1:1, *v*:*v*) solution containing 1.0 M LiTFSI and 0.1 M LiNO_3_. When testing high temperature performances of lithium sulfur batteries, the tetraethylene glycol dimethyl ether/1,3-dioxolane (TEGDME/DOL; 1:1, *v*:*v*) solution containing 1.0 M LiTFSI and 0.1 M LiNO_3_ was served as the electrolyte. The CR2032 coin cell was assembled with the S@CNTs cathode, lithium sheet, separators (the Co_3_O_4_-350/PI/LLZO separator, and the Co_3_O_4_-500/PI/LLZO separator), and the specific electrolyte in an Ar-filled glove box.

The electrochemical impedance spectroscopy (EIS) and cyclic voltammetry (CV) are measured on an electrochemical workstation (ZAHNER-Elek-trik GmbH & Co. KG, Bavaria, Germany). The frequency ranges from 100 kHz to 10 mHz and the voltage amplitude is set at 10 mV. The scan rate is set at 0.5 mV s^−1^. The galvanostatic charge and discharge tests of the as fabricated lithium–sulfur batteries are conducted on a New Wei Lithium Battery Test system within a voltage range of 1.5–2.8 V (vs. Li^+^/Li). The lithium sulfur batteries are placed in a constant temperature oven at 80 °C for high temperature performance tests. The lithium sulfur batteries should rest for more than 4 h before charging and discharging test.

## 3. Results and Discussion

**Figure 1:**
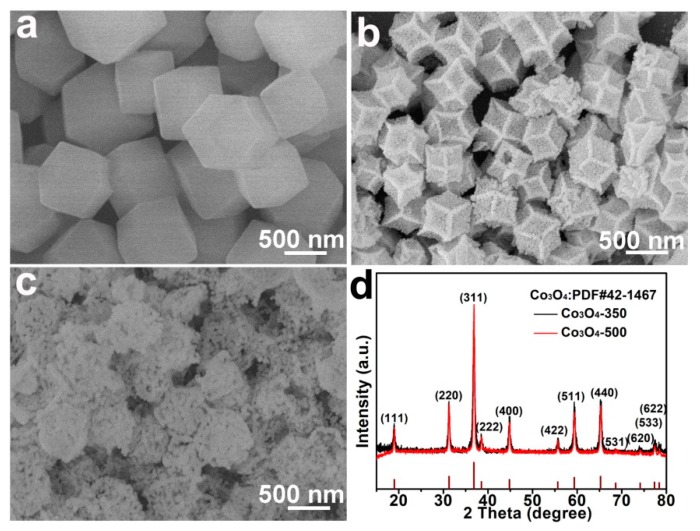
Typical SEM images of the Co-based zeolitic-imidazolate frameworks (ZIF-Co) polyhedron (**a**), the Co_3_O_4_-350 polyhedron (**b**), and the Co_3_O_4_-500 polyhedron (**c**); The XRD patterns (**d**) of both the Co_3_O_4_-350 polyhedron and the Co_3_O_4_-500 polyhedron.

The morphologies and compositions were characterized by typical SEM, TEM, and XRD experiments. Figure 1a displays the SEM image of the ZIF-Co precursors, which show a uniform polyhedron morphology and smooth surface without visible primary particles. The size distribution is shown in Figure S1. After calculation, the average size of the ZIF-Co polyhedrons is determined to be around 650 nm. After facile low-temperature calcination, the surface of the as-prepared Co_3_O_4_-350 polyhedrons (Figure 1b) became rough, and are assembled by primary nanoparticles. The size of the Co_3_O_4_-350 polyhedrons reduced to around 500 nm (Figure S1b), indicating the dimensional shrinkage during the calcination process possibly due to the Ostwald ripening effect [29,30]. It should be noted that calcination temperature plays a vital role in controlling the morphology and size of the Co_3_O_4_ polyhedrons. As shown in Figure 1c, the Co_3_O_4_-500 polyhedrons show creaked morphology with obvious macro pores. The Co_3_O_4_-500 polyhedrons show an average size of 550 nm (Figure S1c) and they are composed of larger-size primary particles. The size distribution (Figure S1) clearly reveals the shrinkage of Co_3_O_4_ polyhedrons after the annealing process. It is easily assumed that the primary nanoparticles in Co_3_O_4_-350 polyhedrons is smaller in size compared to that of the Co_3_O_4_-500 polyhedrons, which indicate the former shows more surface atoms and more grain boundary. These structural merits are beneficial for achieving stronger polysulfide adsorption. In Figure S2, the cross-section SEM image of the Co_3_O_4_-350/PI/LLZO separator reveals that the thickness of the Co_3_O_4_-350 polyhedron coating is about 18 μm, which is sufficient to absorb the soluble polysulfides. The acetylene black and Co_3_O_4_ particle are uniformly distributed throughout the whole coating layer. To accurately determine the thickness of PI film and the LLZO-PEO coating is difficult due to the penetration of partial LLZO-PEO into the large pore of PI film, which is consistent with our previous work [28]. The penetration of LLZO-PEO composite into the PI matrix is beneficial for the blockage of liquid electrolyte leakage and the fast diffusion of soluble polysulfide species.

Figure 1d depicts the XRD patterns of both the Co_3_O_4_-350 polyhedrons and the Co_3_O_4_-500 polyhedrons. All the X-ray diffraction peaks corresponding to various crystalline planes can be well indexed to the standard cubic Co_3_O_4_ (space group Fd-3m, PDF No. 42-1467), indicating that the successful preparation of the crystalline Co_3_O_4_ at the relatively low calcination temperature [31]. The diffraction peaks of both samples are broadened, indicating the small primary particle size. The sharp and intense X-ray diffraction peaks verify the high crystallinity of both samples and no other visible impurity peaks indicate the high purity of the as-prepared samples.

**Figure 2:**
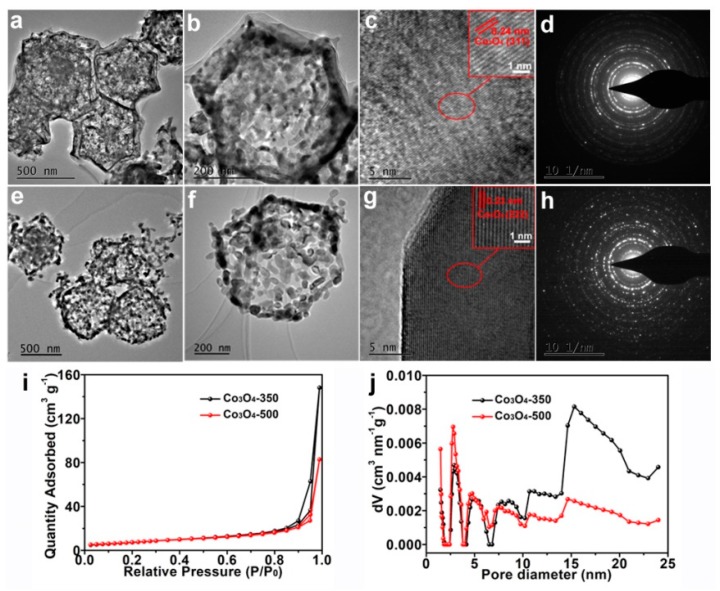
Typical TEM images of both the Co_3_O_4_-350 polyhedrons (**a**,**b**) and the Co_3_O_4_-500 polyhedrons (**e**,**f**); The HRTEM images of both the Co_3_O_4_-350 polyhedrons (**c**) and the Co_3_O_4_-500 polyhedrons (**g**); The SAED pattern of both the Co_3_O_4_-350 polyhedrons (**d**) and the Co_3_O_4_-500 polyhedrons (**h**); N_2_ absorption–desorption isotherms (**i**) and pore-size distributions (**j**) of the Co_3_O_4_-350 polyhedrons and the Co_3_O_4_-500 polyhedrons.

Typical TEM images of both the Co_3_O_4_-350 polyhedrons and the Co_3_O_4_-500 polyhedrons are shown in Figure 2. TEM image in Figure 2a,b presents the hollow and polyhedron features of the Co_3_O_4_-350 polyhedrons. There is an obvious contrast between the dark edge and the gray center of the Co_3_O_4_-350 polyhedrons, indicating the hollow morphology. The average diameter of single polyhedrons is about 500 nm. The small primary nanoparticles with an average diameter of 20 nm are clearly observed. The fringe observed in HRTEM image in Figure 2c corresponds to the interplanar distance of 0.24 nm, which is in good agreement with the lattice spacing of the (311) plane of Co_3_O_4_. In addition, the SAED pattern in Figure 2d illustrates the polycrystallinity of the Co_3_O_4_-350 polyhedrons. While for the Co_3_O_4_-500 polyhedrons, they show an irregular polyhedron morphology with visible crackles as shown in Figure 2e,f. The average diameter of single polyhedron is about 550 nm and much larger primary particles with an average diameter of 40 nm are detected, indicating the growth of primary particles at relatively higher temperature. It should be worth noting that the relatively large primary size of nanoparticles reduces the exposed surface atoms and grain boundary, leading to weak adsorption capability towards soluble polysulfide species. The hollow morphology is also detectable as shown in Figure 2f. The fringe observed in Figure 2g corresponds to the interplanar distance of 0.23 nm, in good agreement with the lattice spacing of the (222) plane of Co_3_O_4_. The SAED pattern in Figure 2g suggests the polycrystalline nature of the Co_3_O_4_-500 polyhedrons. These results unambiguously reveal the successful synthesis of the hierarchical and hollow Co_3_O_4_ polyhedrons with controlled primary nanoparticle size and exposed surface atoms.

The N_2_ adsorption–desorption isotherms of both the Co_3_O_4_-350 polyhedrons and Co_3_O_4_-500 polyhedrons have been shown in Figure 2i, which presents a III-type curve [32]. The hysteresis loop on the adsorption–desorption curves of Co_3_O_4_-350 polyhedrons are relative larger than that of Co_3_O_4_-500 polyhedrons, which indicates more abundant mesopores in the Co_3_O_4_-350 polyhedrons. This might be due to the core-shell structure and the small granular size of the Co_3_O_4_-350 polyhedrons. Through the BET method, the specific surface area are 27.5 m^2^ g^−1^ and 27.1 m^2^ g^−1^ for the Co_3_O_4_-350 polyhedrons and Co_3_O_4_-500 polyhedrons, respectively. As shown in Figure 2j, both samples show hierarchical pore structures. The pore size distribution of the Co_3_O_4_-350 polyhedrons is mainly in the mesopore region, which show an average pore diameter of 15.3 nm. The pore size distribution of the Co_3_O_4_-500 polyhedrons is mainly in the micropore region, which show an average pore diameter of 2.8 nm. The abundant pores together with the relatively large size mesoporous structures will not only be beneficial for the electrolyte wettability and the ion diffusions, but could also be conductive for the adoption and confinement of polysulfide species.

**Figure 3:**
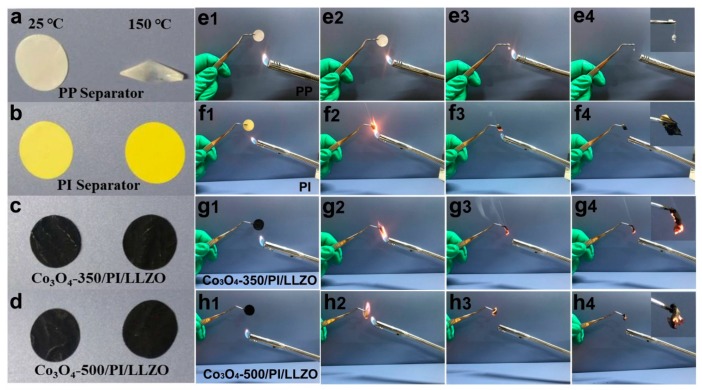
The thermal stability (**a**–**d**) and Flame-retardant properties (**e**–**h**) of the polypropylene (PP) separator, polyimide (PI) separator, Co_3_O_4_-350/PI/LLZO separator and Co_3_O_4_-500/PI/LLZO separator.

The thermal stability is of importance to the safe operation for any battery system. However, the common polyolefin porous separator displays obvious thermal shrinkage, which is the main causation for the thermal runway of typical lithium ion batteries. In order to achieve thermally durable battery systems, the electrospinning polyimide separator is employed due to its high mechanical strength, thermal stability and flame retardance [28]. As shown in Figure 3a, the pristine polypropylene ( PP) separator shows visible dimensional shrinkage at a high temperature of 150 °C for 30 min, while for the other three separators (Figure 3b–d), namely the pristine PI separator, the Co_3_O_4_-350 polyhedron-coating separator, and the Co_3_O_4_-500 polyhedron-coating separator, display no visible shape changes, illustrating the high thermal stability. These results demonstrate the high dimensional stability of Co_3_O_4_-modified PI separators, which guarantees the safe operation of batteries at elevated temperature.

Flame-retardant performances are of equal importance to the safe operation of Li–S battery systems. As for the pristine PP separator, it shows rapid shrinkage and continues to ignite once the flame touches PP separator. And it almost burns completely as shown in Figure 3e. In contrast, there is a self-extinguishing phenomenon for the pristine PI separator, which does not continue to burn after removing the flame. The Co_3_O_4_-modified PI separators show similar experimental results compared to that of the pristine PI separator, illustrating the good flame retardant properties of the as-constructed separators.

**Figure 4:**
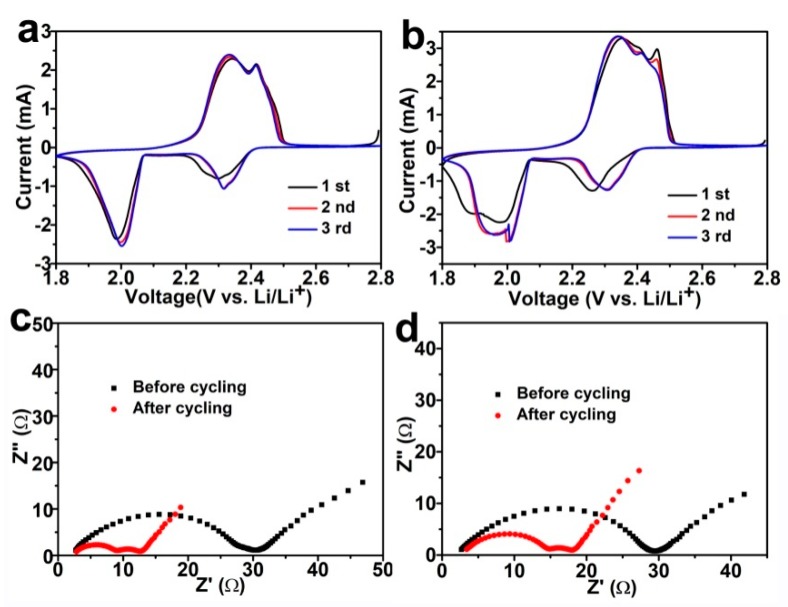
Cyclic voltammetry (CV) curves of Li–S cells using Co_3_O_4_-350/PI/LLZO separator (**a**) and Co_3_O_4_-500/PI/LLZO separator (**b**) at room temperature. Nyquist plots of Li–S cells using Co_3_O_4_-350/PI/LLZO separator (**c**) and Co_3_O_4_-500/PI/LLZO separator (**d**) at room temperature.

Figure 4a presents the CV curves of Li–S cells using Co_3_O_4_-350/PI/LLZO separator, the CV curves display two representative peaks at 2.3 and 2.0 V, which are corresponding to the reduction of sulfur molecular to high-order lithium polysulfides and their further transformation to Li_2_S_2_ and/or Li_2_S, respectively [33,34]. Two characteristic oxidative peaks at 2.3 and 2.4 V could be ascribed to the formations of low-order lithium polysulfides from the final discharged products of Li_2_S_2_/Li_2_S and the high-order lithium polysulfides/sulfur molecular, respectively [14,22]. In addition, the CV curves of the second and third cycles overlap well, indicating a high reversibility and good cycling stability. The CV curves of Li–S cells using the Co_3_O_4_-500/PI/LLZO separator are displayed in Figure 4b, which show similar oxidative/reductive peak characteristics to that of Li–S cells using Co_3_O_4_-350/PI/LLZO separator. The second reduction peak of Li–S cells using the Co_3_O_4_-500/PI/LLZO separator is located at about 1.9 V, indicating a higher voltage polarization and more sluggish electrode kinetic. For comparison, CV curves of Li–S cells using the pristine PI separator are also provided in Figure S3, which show similar oxidative/reductive peak characteristics but with higher voltage polarization. The better conversion kinetics of the Li–S cells using the Co_3_O_4_-350/PI/LLZO separator might be due to the smaller primary granular size, more abundant pore structures and more active sites for adsorbing soluble polysulfides.

Nyquist plots of Li–S cells using the Co_3_O_4_-350/PI/LLZO separator and the Co_3_O_4_-500/PI/LLZO separator are shown in Figure 4c,d, respectively. All EIS curves show two semicircles at high and middle frequencies and one inclined lines at low frequencies, which could be ascribed to the interfacial film resistance, the charge-transfer resistance, and the diffusion resistance in the electrode material, respectively. The equivalent electric circuit from the EIS fitting results is displayed in Figure S4. It is worth noting that the Li–S cells using the Co_3_O_4_-350/PI/LLZO separator deliver lower charge-transfer resistance before (25.4 Ω) and after cycling (6.5 Ω) compared to the Li–S cells using the Co_3_O_4_-500/PI/LLZO separator (26.3 Ω and 8.2 Ω), respectively. The underlying reason of the reduction in resistance after cell cycling might be due to the formation of soluble polysulfide species, which are characterized by faster conversion kinetics compared to the solid insulating sulfur cathode. As for the Li–S cells using the pristine PI separator, both the charge-transfer resistance and the interfacial resistance are much higher than those of the Li–S cells using the as-prepared separators, demonstrating the much sluggish conversion reaction and the low ionic conductivity of the electrolyte-soaked pristine PI separators. This is probably attributed to the smaller primary granular size, more abundant pore structures, and more active sites of the Co_3_O_4_ polyhedron leading to better electrode kinetics for polysulfide conversion.

**Figure 5:**
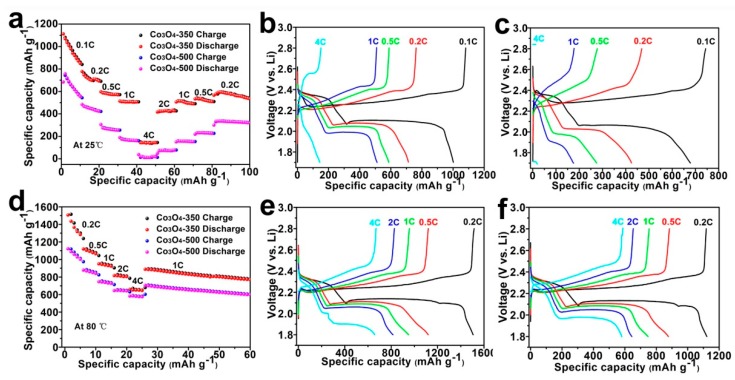
The rate capability of Li–S cell using varied separators at room temperature (**a**) and 80 °C (**d**). The galvanostatic charge/discharge profiles of Li–S cell using Co_3_O_4_-350/PI/LLZO separator at room temperature (**b**) and 80 °C (**e**). The galvanostatic charge/discharge profiles of Li–S cell using Co_3_O_4_-500/PI/LLZO separator at room temperature (**c**) and 80 °C (**f**).

The rate capabilities of Li–S cell using varied separators are tested at room temperature and elevated temperature of 80 °C, which are displayed in Figure 5a,d, respectively. As shown in Figure 5a, Li–S cell using Co_3_O_4_-350/PI/LLZO separator shows much higher specific capacities at varied current densities compared to Li–S cell using Co_3_O_4_-500/PI/LLZO separator. Specifically, when utilizing Co_3_O_4_-350/PI/LLZO separator, room temperature Li–S cells (Figure 5a,b) display high reversible capacities of 1001.8, 712.9, 586.6, 511.1, and 143.3 mAh g^−1^ at current densities of 0.1, 0.2, 0.5, 1.0, and 4.0 C, respectively. When the current density returns to 1.0 C, a reversible capacity of 506 mAh g^−1^ has been achieved, indicating good rate capability. For Li–S cell using Co_3_O_4_-500/PI/LLZO separator, it exhibits relatively lower capacities of 678, 425.1, 274.9, 175.3, and 20 mAh g^−1^ at current densities of 0.1, 0.2, 0.5, 1.0, and 4.0 C, respectively. The galvanostatic charge–discharge profiles of the Li–S cells using the Co_3_O_4_-350/PI/LLZO separator display well-defined voltage plateau at varied current densities as presented in Figure 5b, while for Li–S cell using the Co_3_O_4_-500/PI/LLZO separator (Figure 5c), it shows unambiguous voltage plateaus at high current density and it displays much higher voltage polarizations. These above results indicate the better room temperature electrochemical properties the Li–S cells using the Co_3_O_4_-350/PI/LLZO separator. 

High-temperature durability is of importance to the future practical application of Li–S battery technology. Figure 5d compares the rate performances between Li–S cells using the Co_3_O_4_-350/PI/LLZO separator and the Co_3_O_4_-500/PI/LLZO separator. Clearly, the separator coating of Co_3_O_4_-350 polyhedrons endows Li–S cells with better rate performance. At varied current densities of 0.2, 0.5, 1.0, 2.0, and 4.0 C, they show higher reversible capacities of 1506, 1120, 950, 815, and 658 mAh g^−1^, respectively. When the current density returns to 1.0 C, a high reversible capacity of 887.7 mAh g^−1^ maintains, indicating excellent electrode reversibility. While for Li–S cells using the Co_3_O_4_-500/PI/LLZO separator, they show relatively lower capacities of 1120, 880, 750, 645, and 582 mAh g^−1^ at varied current densities of 0.2, 0.5, 1.0, 2.0, and 4.0 C, respectively. From the galvanostatic charge–discharge profiles for two cells, both of them display well-defined voltage plateau. A slightly lower voltage polarization is detected for Li–S cell using the Co_3_O_4_-350/PI/LLZO separator (Figure 5e,f), which is in consistent with the results from CV curve analyses. All these above analyses unambiguously demonstrate the superior charge storage properties when coating Co_3_O_4_-350 polyhedrons on PI separators, which is attributed to their smaller primary granular size, more abundant pore structures, and more active sites for adsorbing soluble polysulfides.

**Figure 6:**
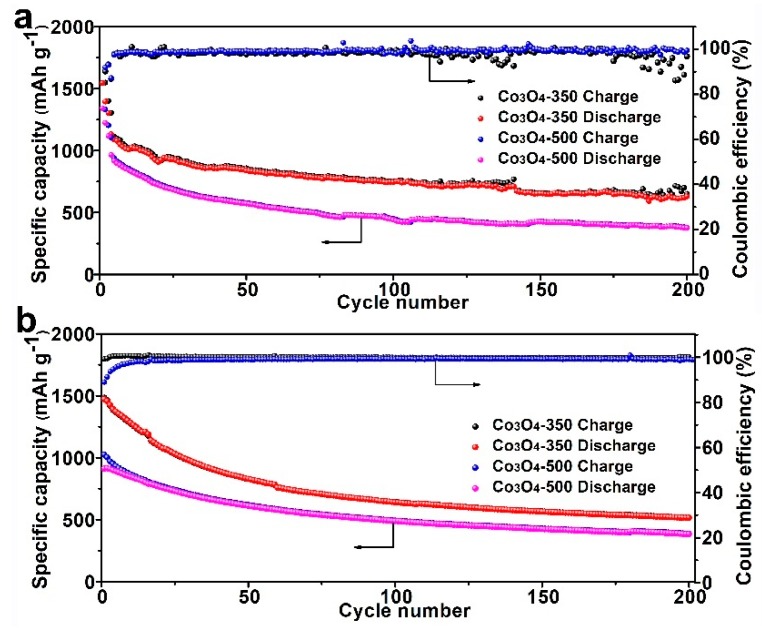
The cycling performances of Li–S cells using varied separator at room temperature (**a**) and 80 °C (**b**).

The room-temperature and elevated temperature cycling performances of Li–S cells using varied separator are tested and shown in Figure 6. At room temperature, Li–S cells using the Co_3_O_4_-350/PI/LLZO separator display an initial high specific capacity of 1132 mAh g^−1^ at a current density of 0.1 C, which gradually decreases to 630 mAh g^−1^ after 200 cycles. While for Li–S cells using the Co_3_O_4_-500/PI/LLZO separator, they show a high initial capacity of 964 mAh g^−1^, which reduce to a relatively lower value of 376 mAh g^−1^ after 200 cycles, indicating the relatively poor cycling properties. It is worth noting that after several initial cycles the Coulombic efficiencies of both cells reach almost 100% during the repeated charge–discharge processes, illustrating the high reversibility. At an elevated temperature of 80 °C, a higher capacity retention of 516 mAh g^−1^ is obtained for Li–S cells using the Co_3_O_4_-350/PI/LLZO separator. These above results clearly reveal the better cycling stability and high temperature durability of the as-constructed Li–S cells using Co_3_O_4_-350/PI/LLZO separator. 

**Figure 7:**
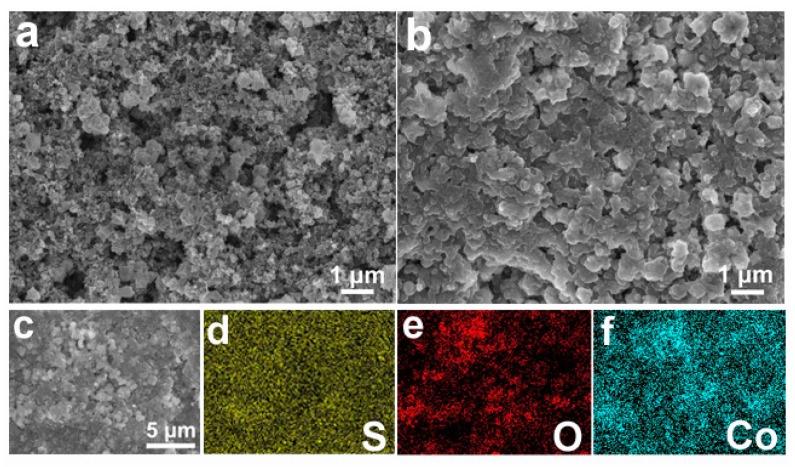
Typical SEM images of the Co_3_O_4_-350/PI/LLZO separator before cycling (**a**) and after cycling (**b**); the corresponding EDS-mapping images of the cycled Co_3_O_4_-350/PI/LLZO separator (**c**: SEM image; **d**: element S; **e**: element O; **f**: element Co).

In order to investigate the polysulfide blockage effect of the Co_3_O_4_-350/PI/LLZO separator, typical SEM images and the corresponding EDS-mapping images have been provided. As for the pristine separator, the Co_3_O_4_-350 polyhedron is homogeneously distributed throughout the whole separator as shown in Figure 7a. After cell cycling, the surface of the Co_3_O_4_-350/PI/LLZO separator is covered by a thin layer consisting of solid-state polysulfide-containing speciation as indicated in Figure 7b. Surprisingly, the polyhedron morphology of the Co_3_O_4_-350 particle does not change even after cell cycling, possessing the enduring effect on physically blocking the soluble polysulfides. The corresponding EDS-mapping images of the cycled Co_3_O_4_-350/PI/LLZO separator as shown in Figure 7c–f indicate that the sulfur-containing species are detected after cell cycling and the Co_3_O_4_-350 polyhedron remains un-pulverized due to the element signal overlap in Figure 7e,f. These results show that the robust and reliable Co_3_O_4_-350 polyhedron on the Co_3_O_4_-350/PI/LLZO separator can effectively block the diffusion of soluble polysulfide species without dramatic microstructure deformation. 

## 4. Conclusions

In summary, a unique Co_3_O_4_ polyhedral coating on thermal-stable polyimide separators was successfully constructed by a simple one-step low-temperature calcination method utilizing metal-organic framework of ZIF-Co precursors. By a comprehensive structural investigation, several structural merits including small primary particle size, large pore size, rich grain boundary, and high ionic conductivity have been revealed, which endow the Co_3_O_4_ polyhedral structures with the ability to adequately adsorb dissolved polysulfides. The flexible-rigid LLZO-PEO coating has been designed on another side of the polyimide non-woven membranes to inhibit the growth of lithium dendrites. The as-fabricated Co_3_O_4_/PI/LLZO composite separators displayed dimensional stability, good mechanical strength, flame retardant properties and excellent ionic conductivity. Our experimental results show that Li–S cells utilizing the Co_3_O_4_-350 polyhedrons coated PI separator display unprecedented rate performance 658 mAh g^−1^ at 4.0 C and cycling stability (516 mAh g^−1^ after 200 cycles) when tested at an elevated temperature of 80 °C, which are much better than the state-of-the-art results. Our present work is believed to encourage more research on the separator engineering for high temperature operation.

## Supplementary Materials

The following are available online at https://www.mdpi.com/2079-4991/9/11/1574/s1, Figure S1: Size distribution; Figure S2: SEM image; Figure S3: CV curve; Figure S4: Equivalent electric circuit.
