# Supplementary material for: MOF-Derived Co3O4 Polyhedrons as Efficient Polysulfides Barrier on Polyimide Separators for High Temperature Lithium–Sulfur Batteries
Source: Nanomaterials (Basel). 2019 Nov 6;9(11):1574. doi: 10.3390/nano9111574 (PMC6915487; doi:10.3390/nano9111574)
Supplement: Supplementary file 1 [file nanomaterials-09-01574-s001.pdf]

Supporting information for

MOF-Derived Co_3O_4 Polyhedrons as Efficient Polysulfides Barrier on Polyimide Separators for High Temperature Lithium–sulfur Batteries

Zhenfang Zhou [1], Yue Li [1], Tingting Fang [1], Yufeng Zhao [1,2,*], Qingjie Wang [3], Jiujun Zhang [2] and Zhongfu Zhou [1,4,*]

[1] School of Materials Science and Engineering, Shanghai University, Shanghai 200444, China; zzf890225@163.com (Z.Z.); liyuejanewatson@163.com (Y.L.); ftt550627949@sina.com (T.F.)
[2] Institute for Sustainable Energy, College of Science, Shanghai University, Shanghai 200444, China; jiujun.zhang@i.shu.edu.cn
[3] State Key Laboratory of Advanced Chemical Power Sources, Guizhou Meiling Power Sources Co. Ltd., Zunyi, Guizhou 563003, China; wqj3401@163.com
[4] Department of Physics, Aberystwyth University, Aberystwyth SY23 3BZ, UK
* Correspondence: yufengzhao@ysu.edu.cn (Y.Z.); z.zhou@shu.edu.cn (Z.Z.)

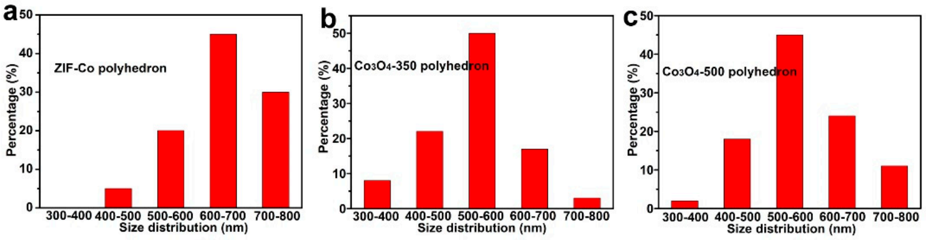

Figure S1. The size distribution of the ZIF-Co polyhedron (a), the Co_3O_4-350 polyhedron (b), and the Co_3O_4-500 polyhedron (c).

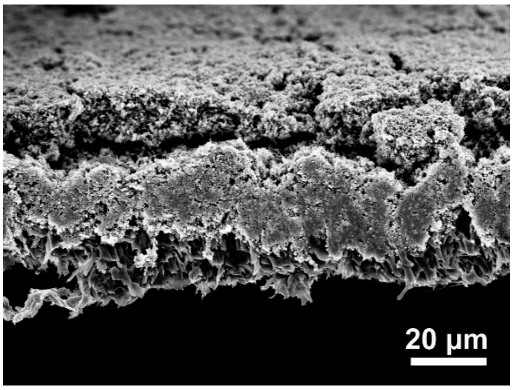

Figure S2. Typical cross-section SEM image of the Co_3O_4-350/PI/LLZO separator.

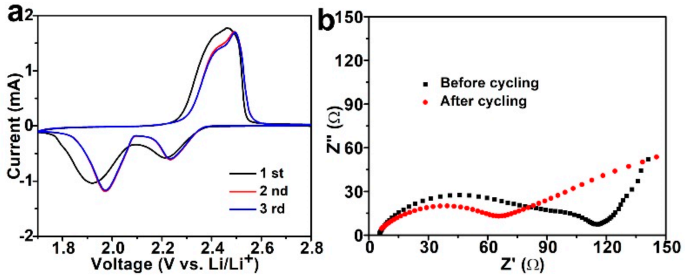

Figure S3. Typical CV curves and Nyquist plots of Li-S cell using the pristine PI separator.

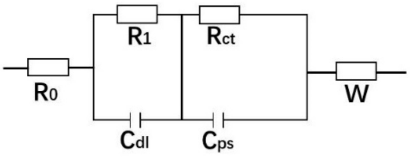

Figure S4. The equivalent electric circuit from the EIS fitting results.
